# Perianal Crohn-like disease in infancy revealing multisystem Langerhans cell histiocytosis

**DOI:** 10.1186/s12887-026-06907-2

**Published:** 2026-04-29

**Authors:** Buket Daldaban Sarıca, Meriban Karadoğan, Ramazan Arslan, Alper Sayıner, Atıl Avcı

**Affiliations:** 1 Department of Pediatrics, Division of Gastroenterology, Hepatology and Nutrition, University of Health Sciences, Kayseri City Hospital, Kayseri, Türkiye; 2 Department of Pediatrics, Division of Hematology and Oncology, University of Health Sciences, Kayseri City Hospital, Kayseri, Türkiye; 3 Department of Pathology, University of Health Sciences, Kayseri City Hospital, Kayseri, Türkiye; 4 Department of Dermatology, University of Health Sciences, Kayseri City Hospital, Kayseri, Türkiye

**Keywords:** Very early-onset inflammatory bowel disease, Perianal lesions, *BRAF*

## Abstract

**Background:**

Langerhans cell histiocytosis (LCH) is a rare inflammatory myeloid neoplasm characterized by abnormal myeloid differentiation and activation of the mitogen-activated protein kinase (MAPK) pathway. Since atypical dermatological or gastrointestinal manifestations in early childhood can lead to diagnostic delays, timely recognition of this potentially multisystemic disease is critical for a favorable prognosis.

**Case presentation:**

A 22-month-old male patient presented to the pediatric gastroenterology outpatient clinic with complaints of bloody-mucoid diarrhea for three months, significant weight loss, and non-healing ulcerative lesions in the perianal region. His family history included an uncle with ulcerative colitis.

A preliminary diagnosis of very early-onset inflammatory bowel disease (IBD) was made, and upper and lower gastrointestinal endoscopy was performed. Histopathological evaluation of the lower gastrointestinal tract revealed nonspecific inflammatory changes. Stool calprotectin level was 144 µg/g. The presence of seborrheic dermatitis-like lesions on the scalp suggested the possibility of LCH, and previous pathology samples were re-examined. There was no immunohistochemical staining for Langerhans cell markers in bowel biopsies, but strong positivity was detected in perianal biopsy samples. Immunohistochemical analysis revealed positive BRAF expression. Imaging to assess systemic involvement revealed a contrast-enhancing mass lesion on pituitary magnetic resonance imaging. A staging study was performed to determine risk organ involvement. Given the absence of liver, spleen, and bone marrow involvement, the disease was classified as risk organ-negative multisystem LCH (RO-MS-LCH).

**Conclusion:**

This case highlights the need to consider multiple systemic disorders, particularly Langerhans cell histiocytosis (LCH), in children presenting with gastrointestinal and perianal symptoms suggestive of inflammatory bowel disease (IBD), especially in the presence of atypical skin lesions and the absence of histopathological confirmation. Early and comprehensive clinical evaluation plays a key role in preventing diagnostic delays and initiating appropriate treatment in a timely manner.

## Background

Langerhans cell histiocytosis (LCH) is a rare inflammatory myeloid neoplasm with a broad clinical spectrum characterized by aberrant myeloid differentiation and mitogen-activated protein kinase (MAPK) pathway activation. LCH exhibits clinically diverse organ involvement, particularly in childhood [1]. The clinical presentation of the disease ranges from single-system involvement to multiple organ failure, with multisystem disease—especially when high-risk organs are affected—being associated with a more aggressive course and greater morbidity [2]. As atypical dermatological or gastrointestinal manifestations in early childhood may result in diagnostic delays, timely recognition of potential multisystemic disease is critical for a favorable prognosis [3].

Here, we report a case of a 22-month-old male who initially presented with gastrointestinal and cutaneous findings suggestive of inflammatory bowel disease (IBD) but was later diagnosed with low-risk multisystem LCH after further evaluation.

## Case presentation

**Fig. 1:**
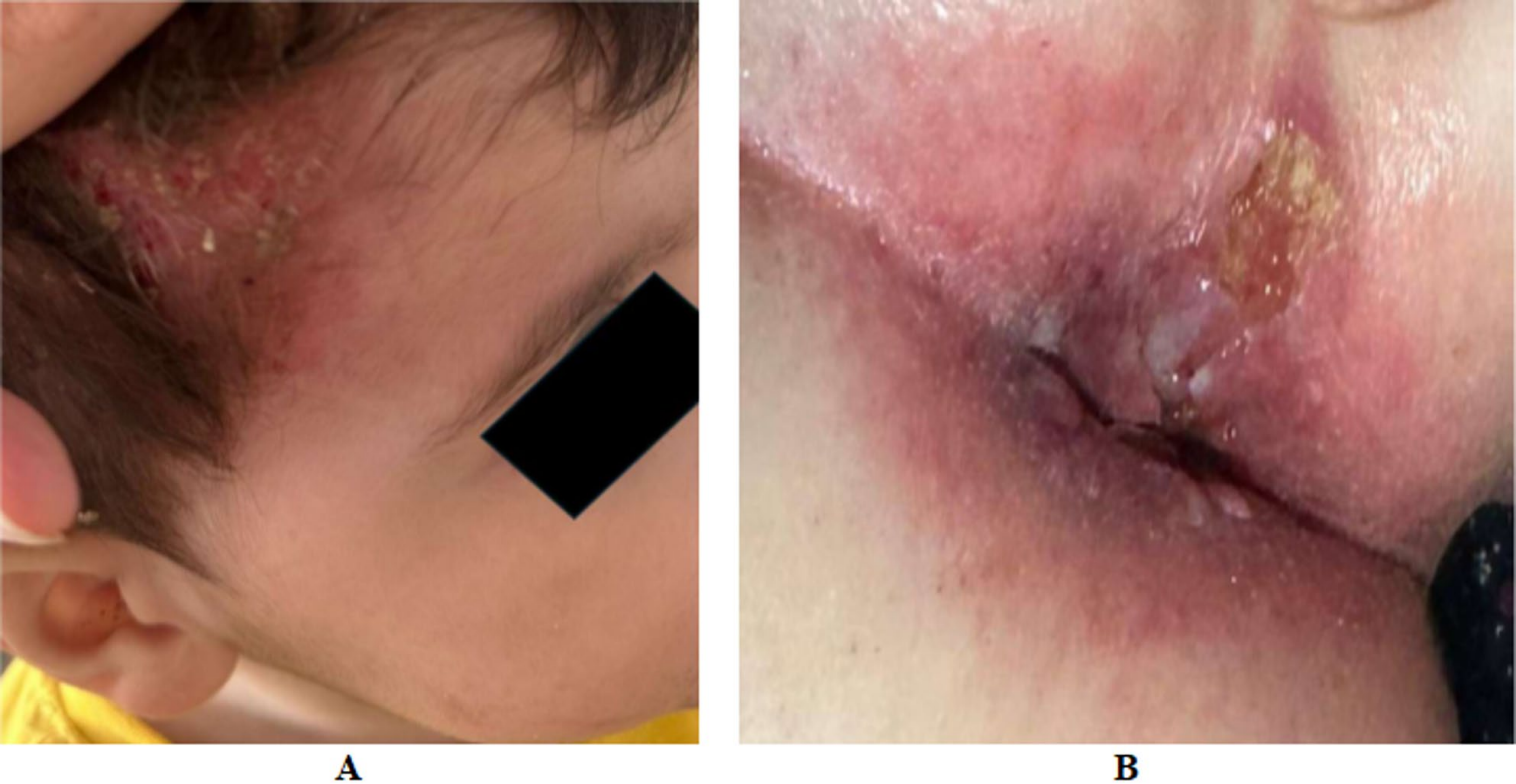
Cutaneous and mucosal LCH presenting as (**A**) Scalp rash and (**B**) Perianal lesion

A 22-month-old male patient presented to the pediatric gastroenterology outpatient clinic with a three-month history of bloody-mucoid diarrhea, significant weight loss, and non-healing ulcerative lesions in the perianal region. His clinical history was unremarkable, whereas his family history was significant for ulcerative colitis in his uncle. In addition to perianal involvement, the family reported erythematous and scaly scalp rashes suggestive of seborrheic dermatitis (Fig. 1).

Based on the early onset of perianal disease, mild elevation of acute-phase reactants, and a positive family history, a preliminary diagnosis of very early-onset IBD was made, and upper and lower gastrointestinal endoscopy was performed. Endoscopic examination revealed macroscopically normal mucosa; however, histopathological evaluation of biopsy samples identified nonspecific inflammatory changes. The fecal calprotectin level was 144 µg/g. Laboratory investigations showed normal liver enzymes and a hemoglobin level of 13.3 g/dL, a white blood cell count of 8900/mm³, and a platelet count of 344,000/mm³.

In view of the early presentation of perianal disease, other potential conditions that may mimic Crohn disease in infancy were systematically investigated. Microbiological analyses of stool samples ruled out parasitic, bacterial and viral infections. Given the early age at diagnosis, monogenic IBD and primary immune disorders were also evaluated. Baseline immunological testing, comprising immunoglobulin levels and lymphocyte immunophenotyping, identified no findings suggestive of underlying immune dysfunction. The lack of evidence for systemic infection, immunodeficiency, or monogenic early-onset IBD suggested a non-IBD origin of the perianal lesions.

**Fig. 2:**
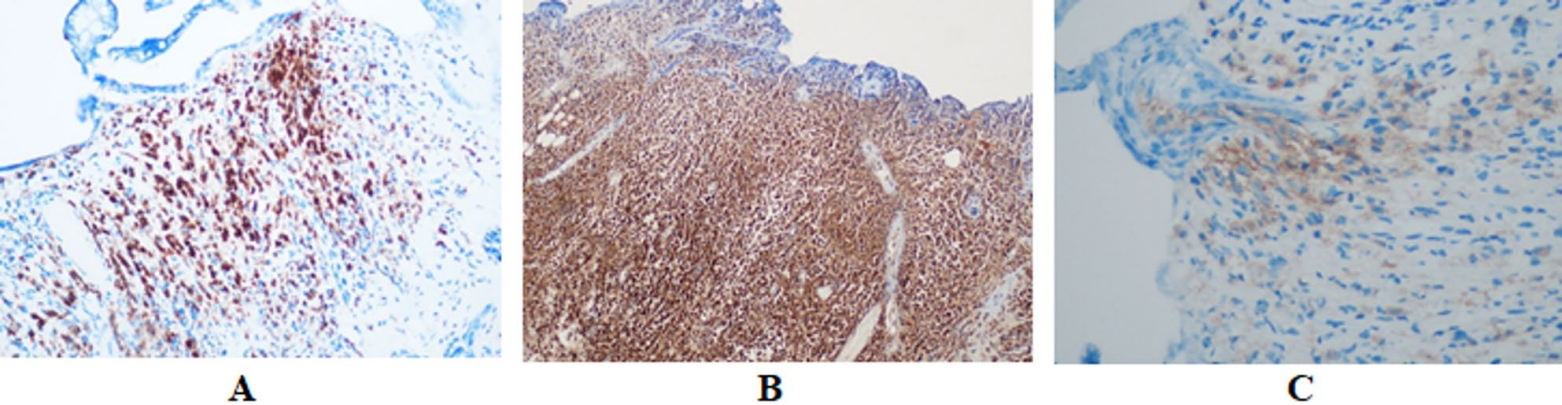
Histopathological images of perianal LCH lesion. **A** Diffuse immunohistochemical staining with Langerin. **B** Positive S-100 immunohistochemical staining. **C** Focal membranous staining with CD1a. Histopathological findings are shown at 200x magnification

Given the nonspecific clinical findings and ongoing diagnostic uncertainty, the differential diagnoses were re-evaluated. The presence of seborrheic dermatitis–like lesions, particularly on the scalp, suggested the possibility of LCH, and prior pathology samples were re-examined. Immunohistochemical staining for Langerhans cell markers was absent in the intestinal biopsies but strongly positive in the perianal biopsy specimens (Fig. 2). Immunohistochemical analysis revealed positive *BRAF* expression.

**Fig. 3:**
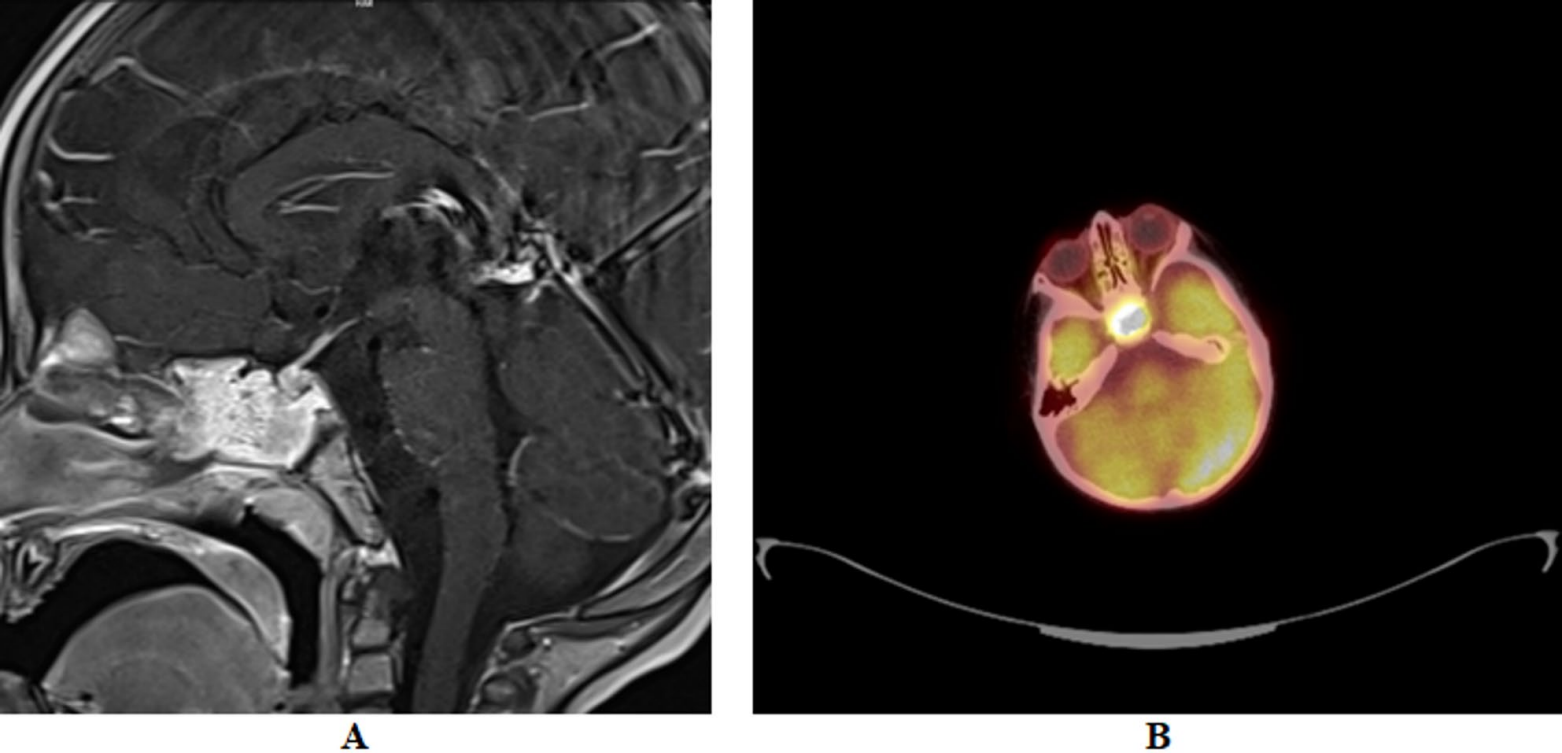
Radiological findings. **A** Magnetic resonance imaging (MRI) showing hypointense pituitary mass on contrast T1-weighted imaging. **B** Positron emission tomography (PET) demonstrating hypermetabolic activity with a focus of increased FDG uptake

Imaging performed to assess systemic involvement revealed a contrast-enhancing mass lesion measuring 17 × 30 × 20 mm, extending from the clivus to the base of the sphenoid bone and the ethmoid region on pituitary magnetic resonance imaging (Fig. 3A). Positron emission tomography revealed significant hypermetabolic activity in the pituitary fossa (19 × 17 mm; Fig. 3B), supporting the presence of multisystem involvement.

Based on integrated clinical, histopathological, and radiological findings, a diagnosis of LCH was confirmed. The staging workup was conducted to determine risk-organ involvement. Abdominal ultrasonography showed normal liver parenchymal appearance, and normal spleen size. Peripheral blood counts were within normal limits for age, and bone marrow aspiration and biopsy demonstrated normocellular trilineage hematopoiesis without evidence of histiocytic infiltration. Considering the absence of liver, spleen and bone marrow involvement, the disease was categorized as risk-organ negative multisystem LCH (RO − MS-LCH).

In view of the detection of a CNS-risk lesion located in the clivus–sphenoid region, a detailed hypothalamo–pituitary endocrine axis assessment was conducted. Serum electrolytes, plasma and urinary osmolality, and monitoring of clinical fluid balance showed no signs of central diabetes insipidus. Initial endocrine evaluation including thyroid function parameters, serum morning cortisol, and growth indices were within normal reference ranges. No clinical or biochemical indicators of pituitary hormone insufficiency were found at diagnosis. The patient remains under scheduled endocrine monitoring owing to the risk for subsequent hypothalamo–pituitary impairment.

Systemic therapy with vinblastine (6 mg/m² weekly) and prednisolone (40 mg/m²/day with standard tapering) was initiated according to current LCH treatment guidelines for RO− multisystem disease [4]. The patient remains on protocol-based treatment, with follow-up showing significant regression of perianal and cutaneous lesions, improvement in gastrointestinal symptoms, and marked overall clinical recovery. Due to the presence of a CNS-risk lesion, the patient is under close monitoring, particularly for the development of diabetes insipidus.

## Discussion

This case underscores the importance of considering rare systemic diseases in the differential diagnosis of patients presenting in early childhood with gastrointestinal and perianal findings suggestive of IBD. The patient’s clinical features including chronic diarrhea, perianal ulcerative lesions, mildly elevated fecal calprotectin, and a positive family history initially supported a diagnosis of very early-onset IBD. Although fecal calprotectin is commonly used as a biomarker of intestinal inflammatory activity, it has limited specificity in infants and early childhood. Increased levels may occur in a wide range of inflammatory, infectious, and tissue-infiltrating disorders beyond IBD [5]. In the present case, the mild elevation in fecal calprotectin levels likely represented nonspecific intestinal inflammation rather than underlying intestinal IBD, underscoring the need for careful interpretation in young children. This is particularly important in infants and young children, in whom fecal calprotectin lacks specificity and may be elevated even in the absence of true IBD.

However, the absence of macroscopic pathology on endoscopic examination and the presence of only nonspecific inflammatory changes in intestinal biopsies precluded histopathological confirmation of intestinal inflammation. Moreover, the presence of widespread seborrheic dermatitis–like scalp lesions raised the suspicion of systemic disease. Consequently, the clinical evaluation was broadened from an organ-specific approach to a multisystem perspective, and histopathological examination of the perianal lesion confirmed the diagnosis of LCH.

LCH is a rare clonal myeloid cell neoplasm driven by MAPK pathway activation, with inflammatory features and a heterogeneous clinical spectrum [1]. *BRAF* mutations are the most common drivers of MAPK signaling pathway activation in LCH, as seen in our patient.

Multisystem LCH is classified based on the presence of risk-organ involvement, including the liver, spleen and bone marrow [6]. Gastrointestinal involvement in LCH is uncommon, reported in approximately 2% of cases, and usually occurs in the context of multisystem disease [7]. When present, particularly in the presence of risk-organ involvement, it is associated with poor prognosis and increased mortality rates estimated up to 60% [8]. Endoscopic and histopathological findings are often nonspecific and diagnosis is established by demonstrating histiocytic infiltration in the lamina propria and CD1a and CD207 (langerin) immunopositivity [9]. Although central nervous system (CNS) involvement was present, the absence of liver, spleen, or bone marrow involvement led to the classification as RO − MS-LCH. While the RO − MS-LCH is generally associated with a more favorable prognosis [10], CNS risk lesions including sphenoid, mastoid, orbit, clivus, or temporal bones, as well as ocular involvement remain clinically significant due to their association with hypothalamo-pituitary axis dysfunction, permanent endocrine sequelae such as diabetes insipidus and neurodegenerative complications [10].

This case demonstrates that multisystem LCH may clinically resemble very early-onset IBD, especially when perianal manifestations are pronounced. Systemic staging assessment, site-specific biopsy from involved tissues, and periodic endocrine follow-up are critical for precise classification and ongoing management.

## Conclusion

In conclusion, this case underscores the need for considering multisystem diseases, particularly LCH, in children presenting with gastrointestinal and perianal manifestations suggestive of IBD, in the presence of atypical cutaneous lesions and in the absence of histopathological confirmation. Diagnostic delays often reflect the rarity of the disease, the lack of standardized diagnostic criteria, and the misinterpretation of gastrointestinal symptoms as isolated digestive pathology. Early and comprehensive clinical evaluation plays a key role in preventing diagnostic delay and enabling timely initiation of appropriate treatment.

## Data Availability

No datasets were generated or analysed during the current study.

## Abbreviations

CNS: Central nervous system
IBD: Inflammatory bowel disease
LCH: Langerhans cell histiocytosis
MAPK: Mitogen-activated protein kinase
RO-MS-LCH: Risk organ-negative multisystem LCH
